# Finding the way - nursing staff’s perceptions and experiences of outdoor walks with residents in dementia care homes: a qualitative study

**DOI:** 10.1186/s12912-025-03771-w

**Published:** 2025-09-01

**Authors:** Cecilia Sjöholm, Kirsti Skavberg Roaldsen, Paul A. Gardiner, Ing-Mari Dohrn

**Affiliations:** 1 Department of Elderly Care and Social Services, City of Gothenburg, Gothenburg, Sweden; 2 Department of Neurobiology, Care Sciences and Society, Karolinska Institutet, Stockholm, Sweden; 3 School of Public Health, The University of Queensland, Brisbane, Australia

**Keywords:** Dementia, Nursing care, Physical activity, Qualitative, Walking

## Abstract

**Background:**

Outdoor walks have been shown to provide physical and cognitive benefits and may help reduce behavioral and psychological symptoms of dementia (BPSD) in care home residents. However, little is known about how nursing staff perceive and integrate these walks into daily care. This study aimed to explore staff perceptions and experiences of outdoor walks, focusing on their benefits and how to integrate them into routine care.

**Methods:**

Semi-structured interviews were conducted with seven informants, all nursing staff, from six dementia care homes located in different residential areas in Sweden. Data were analyzed using qualitative content analysis.

**Results:**

Three main categories were identified: ‘Walks with purpose and meaning’, ‘Challenges with outdoor walks and ways to deal with them’, and ‘Emotional, physical and relational responses to outdoor walks’. Outdoor walks were perceived valuable for residents, staff, and relatives from multiple perspectives. Informants consistently described outdoor walks as meaningful and beneficial for residents, contributing to improvements in physical, psychological, cognitive, social, and existential well-being, both during and after the walks. However, walks could occasionally increase anxiety afterward. Challenges were described both in the preparation process and during the walk, such as stressful environments, weather conditions, and varying individual health conditions and needs. Insufficient staffing, competing tasks, differing views among staff regarding the importance of walking, and the absence of established routines for walks were also mentioned. The informants had developed various a person-centered strategies to manage many of these challenges and risks, based on their knowledge and experience in dementia care.

**Conclusion:**

Outdoor walks were highly valued by staff and could be integrated into daily dementia care with appropriate planning. A wide range of health benefits were described, including reduced BPSD, suggesting that walks may help reduce the need for medication. Moreover, outdoor walks were viewed as meaningful activities that fostered social connection and offered residents a way to engage with nature and the surrounding community.

**Trial registration:**

Not applicable.

**Supplementary Information:**

The online version contains supplementary material available at 10.1186/s12912-025-03771-w.

## Background

The prevalence of people with dementia is rising both in Sweden and globally. In 2019, it was estimated that 57 million people worldwide were living with dementia, a number expected to increase to 152 million by 2050 [1]. In Sweden, approximately 144,000 people over 65 years were diagnosed with dementia in 2019, and it was estimated that 35–40% of these individuals reside in nursing homes [2].

Common symptoms of dementia include impaired memory, language difficulties, mood swings, impaired judgment, and lack of initiative [3], which are severe enough to impair the ability to perform daily activities. The disease also leads to reduced physical function, balance, and walking ability [4], which increases the risk of falls [5]. Additionally, individuals with dementia are at heightened risk of developing behavioral and psychological symptoms of dementia (BPSD), such as agitation, depression, apathy, psychosis, sleep problems and wandering [6]. While there is no curative treatment for dementia, both the World Health Organization (WHO) and the Swedish National Board of Health and Welfare emphasize the importance of physical activity, as well as engaging in stimulating activities and social interactions, to support brain function, maintain daily functioning, and promote quality of life and well-being [7, 8]. Carers of people with dementia should adopt a person-centered approach that focuses on the individual rather than the diagnosis. This involves trying to understand what is best for each individual from their perspective, involving the person in decision-making, and maintaining a meaningful relationship with them [7]. A written life story supports this by offering insight into who the person is and has been, helping staff connect and provide more personalized care [7].

Numerous studies demonstrate that physical activity benefits individuals with dementia by improving mobility, gait speed, strength, balance, and endurance [9, 10]. Physical activity can also reduce fall risk, enhance the ability to perform activities of daily living, and support cognitive functions such as executive function and memory [11–13]. For example, older adults diagnosed with mild-to-moderate Alzheimer’s disease who walked for 1 h/week experienced a stabilization in cognitive functioning after one year and those who walked for 2 h/week or more showed a significant improvement [14].

Walking is a simple and cost-effective way for older adults to stay physically active. It can be easily adapted to an individual’s capabilities, making it particularly suitable for people with dementia. Walking also provides an opportunity for them to engage with their surroundings, especially in nature. According to a systematic review and meta-analysis, group walks produce several positive health effects, such as reduced blood pressure, cholesterol, and body mass index, as well as a decreased risk of depression [15]. However, research shows that both community-dwelling individuals with dementia and those living in residential care homes are mostly sedentary, engaging in less physical activity than healthy peers [16, 17]. In nursing homes, residents with dementia often depend on others to go outdoors due to difficulties initiating activities, mobility issues, fear of falling, or disorientation [18]. It has been shown that residents in dementia care homes are physically inactive for 72% of the day [17].

Recent research has highlighted the numerous health benefits of spending time in nature, including reduced stress, improved sleep, lower anxiety, decreased depression, and enhanced well-being [19]. Nature-based activities for older persons show similar positive effects, offering sensory stimulation, boosting physical and cognitive functions, enhancing self-esteem, and providing meaningful social engagement [19, 20]. Individuals with mild Alzheimer’s disease have emphasized the importance of nature for achieving health and a sense of well-being when engaging in physical activity, such as walking or cycling [21, 22]. Even so, dementia symptoms can make it challenging for individuals to access and enjoy nature [23].

Several studies have shown that structured walks, both indoors and outdoors, can benefit residents with dementia by helping maintain functional mobility, improving physical function, activities in daily living, and quality of life, and potentially slowing cognitive decline [24–26]. In one study that used a person-centered approach, a walking intervention was supported by an individualized care plan that incorporated elements such as the resident’s biography, communication strategies, engagement preferences, behavioral cues, and mobility support [24]. Additionally, some studies report that outdoor walking programs may help reduce wandering behavior and nighttime restlessness [27, 28].

Given the potential health benefits of outdoor walks for people with dementia, it is important to increase our understanding of how these walks can be implemented into daily routines in dementia care homes. While previous studies have focused on structured walking programs within research settings [24, 26–28], there is a lack of research exploring nursing staff’s perceptions and experiences of facilitating outdoor walks as part of routine care practice.

## Methods

### Aim

The aim of the study was to explore the nursing staff’s perceptions and experiences of outdoor walks with residents in dementia care homes, focusing on perceived benefits and how to facilitate integrating walks into regular care routines.

### Recruitment and informants

Recruitment started by emailing managers at 12 dementia care homes in the Gothenburg area that, according to their websites, offered outdoor walks or outdoor activities. Additionally, one dementia care home outside Gothenburg was contacted due to their extensive experience with outdoor walks as part of regular care. The email included information letters for both unit managers and nursing staff. After receiving no responses within a week, the same message was sent to the managers of 26 additional dementia care homes in Gothenburg and to 13 physiotherapists in the Cognitive Diseases Network for physiotherapists in Gothenburg. The first author also reached out by phone to three of the managers that were previously known to her.

A final sample of seven informants was recruited from six dementia care homes located in different residential areas. These homes varied in size, accommodating 32 to 125 total residents, with 5−8 residents per unit. Residents’ ages ranged from 55 to 105 years. All informants were female nursing staff aged 29 to 59 years (median: 40), with workplace experience ranging from 3 to 23 years (median: 10), relevant to the study’s purpose. Four informants were of ethnic Swedish origin, while three were of non-Swedish ethnic backgrounds. For additional background characteristics, see Table 1.

**Table 1:** Background characteristics of the informants

Informant	Profession	Workplace experience, years	Dementia home area
1	Assistant nurse	7	Urban
2	Assistant nurse	21	Urban
3	Assistant nurse	10	Urban
4	Care assistant	4	Urban
5	Assistant nurse	22	Rural
6	Assistant nurse	3	Suburban
7	Assistant nurse	23	Suburban

All informants signed an informed consent form and were given both oral and written information regarding the study’s purpose and the right to withdraw at any time without providing a reason. The study was conducted in accordance with the Declaration of Helsinki and participation was approved by the managers of the dementia care homes.

### Data collection

Individual semi-structured interviews were selected for the capacity to generate rich, detailed data [29]. An interview guide with open-ended questions was developed for this study to allow informants to express their experiences and views in their own words [29]. The guide included both general and specific questions related to the purpose of the study. A pilot interview, not included in the study, was conducted with a colleague of the first author to test the questions and refine interview techniques. No changes were made to the guide following the pilot interview. (An English language version of the interview guide can be found in Supplementary file Appendix 1).

All interviews were conducted in March−April 2023 by the first author, a physiotherapist with extensive experience in dementia care, which facilitated deeper follow-up on issues raised by informants, enriching the interviews. Two interviews were conducted digitally via Microsoft Teams, while the remaining interviews took place at the informants’ workplaces. The interviewer had no prior relationship with the participants. Interviews were recorded and lasted 19–49 min. Recordings were transcribed verbatim using the transcription function in Microsoft Word 365 and subsequently proofread and adjusted by the first author.

### Data analysis

The analysis was guided by the study aim and the transcripts were systematically analyzed following the stages in qualitative content analysis with an inductive approach [30, 31]. Initially, the transcripts were read multiple times to gain an overall understanding of the data. Relevant sections were identified and reduced into approximately 700 meaning units. These units were then condensed and assigned descriptive codes that captured their essential content [30, 31]. Related codes sharing a common theme or idea were first grouped into subcategories and subsequently organized into categories to reflect broader themes [30, 31]. Examples illustrating the analysis process are provided in Table 2. To enhance credibility, the analysis was conducted by the first and the last author, with iterative review of the stages to ensure no significant information was overlooked.

The analysis process adhered to criteria ensuring credibility, dependability, and transferability to maintain the quality and trustworthiness of the findings [32]. This included careful attention to detail, consistent methodology, and consideration of how findings could be applied to other contexts. Both the interviews and data analysis were conducted in Swedish. Quotes to demonstrate the categories and subcategories were translated into English by the last author and thereafter critically reviewed and verified by the other authors. All authors participated in reviewing and discussing the analysis and results. The authors’ combined expertise in qualitative research, physical activity, and dementia care greatly contributed to a comprehensive understanding and nuanced interpretation of the data. In addition, the Consolidated Criteria for Reporting Qualitative Studies (COREQ) checklist was followed to ensure the quality of reporting [33].

**Table 2:** Example of the analysis process from meaning unit to category

Meaning unit	Condensed	Code	Subcategory	Category
*To be able to offer some activity*,* some physical activity. (I6).*	Offer physical activity	Physical activity	Reasons for outdoor walks	Walks with purpose and meaning
*It’s also important to plan so that you know that there are staff on that floor. So that you don’t… so the person there doesn’t feel alone. (I4)*	It is important that there are staff left on each floor and that that person does not feel alone.	Adequate staffing	Impeding and facilitating factors within the organization	Challenges with outdoor walks and ways to deal with them
*It’s a 1*,* if you are anxious*,* or a 2; and a 5 when you are completely satisfied and harmonic. So*,* we did it a week before we started the walks. And of those we selected*,* no one rated a five. But the days we went out for walks*,* they had fives. Completely satisfied. And it lasted for a while after. It wasn’t just during the time we were out*,* it lasted afterwards as well. (I6)*	1 is anxious and 5 is completely satisfied and harmonic. We rated before we started the walks. None of those we chose rated a five. On the days we walked, they rated fives. Completely satisfied. And it lasted for a while, not just during the time we were out.	Become calmer	Benefits and emotional shifts in residents	Emotional, physical and relational responses to outdoor walks

## Results

The analysis resulted in three main categories formed by nine subcategories. The main categories were: ‘Walks with purpose and meaning’, ‘Challenges with outdoor walks and ways to deal with them’, and ‘Emotional, physical and relational responses to outdoor walks’ (Table 3).

**Table 3:** Overview of the results: main categories and subcategories

Main categories	Subcategories
Walks with purpose and meaning	Reasons for outdoor walks
	Creating added value
Challenges with outdoor walks and ways to deal with them	Impeding and facilitating factors within the organization
	Importance of the external environment
	Meeting the individual’s conditions and needs
	Motivation and guidance
Emotional, physical and relational responses to outdoor walks	Benefits and emotional shifts in residents
	Ambivalent experiences among staff
	Emotional relief for family members

### Main category 1: Walks with purpose and meaning

The purpose of walks in dementia homes might influence both their outcomes and the staff’s motivation to engage in them. Informants described how the walks served various functions, including enhancing health through physical activity and outdoor exposure, as well as facilitating social interaction and sensory experiences. Having a specific goal that added value also enhanced the meaningfulness of the walk.

#### Reasons for outdoor walks

The informants indicated a variety of reasons for taking walks with residents. These included general reasons, such as the belief that everyone has a right to spend time outdoors, that physical activity is beneficial for health, and that most people appreciate connecting with nature. There were also reasons more specific to dementia home residents, including that walks can have a calming effect, reduce sedentary time and increase physical activity, help maintain physical abilities, provide a change of environment, and reduce the feeling of being trapped.

*Inmates in prisons have the right to be outside. Animals have a right to be outside. Children in preschool*,* [have the] right to be outside. But not older people. (I6)*

*To be able to feel nature. Being allowed to go and touch a tree. Being able to hold a pinecone while walking. Picking chestnuts*,* picking flowers*,* whatever… To get out and feel. (I4)*

#### Creating added value

Several ways to make the walks pleasant and create added value were mentioned. Ways to enhance the walking experience included capturing the resident’s comments during the walk to start a conversation or using the opportunity for sensory stimulation by smelling flowers, listening to the sounds of nature, or walking on different surfaces. The walks could also provide an opportunity to connect with the community outside the dementia home and foster a sense of belonging, such as stopping to talk to neighbors, playing with children at the playground or buying a cup of coffee or an ice cream at a convenience store. Additionally, it was suggested to involve the resident in choosing the destination, to consider walking in a group, and to plan the walk as an excursion.

*Or I might say: ‘Let’s go to the chickens; we have benches where we can sit.’ And then it’s nice to have coffee out there in the forest*,* to drink something and such. (I2)*

*It doesn’t cost anything. And there’s so much to gain from it. (I5)*

### Main category 2: Challenges with outdoor walks and ways to deal with them

Walks in dementia care homes differ from those in nursing homes in several ways. Challenges arise both in the preparation process and during the walk itself, necessitating active support from staff. Through their experience of walking with people with dementia, the informants had developed various strategies to manage many of these challenges and risks.

#### Impeding and facilitating factors within the organization

Several informants encountered various obstacles within their organization. Examples included lack of time, insufficient staffing, high workload, competing tasks, differing views among staff regarding the importance of walking, and the absence of established routines for walks.

*They are happy just to get them out of bed*,* do the hygiene and feed them. They don’t have the time [for walks]. (I2)*

*I know that some staff don’t like going outside. But at the same time*,* I think this is part of our job too. We’re not just here to serve food*,* to do personal care*,* to do this and that. This is something that has to be done too. (I5)*

The fact that the Social Services Act does not guarantee the right to go outdoors was also seen as a hindrance. Informants mentioned that they could walk with one or, at most, two residents, depending on the level of support required. Strategies for addressing these challenges included taking shorter walks, assisting each other across departments, being flexible with tasks, utilizing help from interns, and selecting times of day with the most staffing.

*You’re flexible. You save those tasks for the afternoon. You check if the weather is good in the morning: ’Well*,* I can postpone the other task’. […] That way you have the opportunity to go out in the morning. (I2)*

One approach was to carry a phone in case of unforeseen incidents requiring assistance from colleagues. Another was to bring up issues related to walks during team meetings.

#### Importance of the external environment

The external environment played a crucial role in the walking experience and could present challenges. Several informants emphasized the value of a calm setting, noting that noisy, high-traffic areas negatively affected residents and increased safety risks. Those working in urban areas highlighted the impact of heavy traffic, while staff in suburban or rural settings appreciated access to quieter, low-traffic areas perceived as safer and less stressful for residents.

*I walk on small streets. Streets that I know*,* without cars or crosswalks. Large crosswalks*,* no*,* that’s not possible. (I3)*

The weather could also be challenging due to the risk of slipping or sunburn. Having different walking routes and seating options, as well as asphalt walkways − especially for those using walkers − were mentioned as helpful. Nice weather made it easier to motivate residents to go for a walk. Strategies included choosing quiet, traffic-free environments, walking indoors during bad weather, ensuring residents had good shoes and appropriate clothing, using hats for sun protection, and bringing water in warm weather. One informant expressed a wish for new homes for older people to be situated in walkable environments.

*And to the city and everyone involved in building care homes for older people: Make sure you consider the location! […] If there’s a courtyard or the surrounding space. Use it to create an inviting environment that encourages walking. (I4)*

#### Meeting the individual’s conditions and needs

Another challenge was accommodating the individuals’ varying conditions and needs. Factors such as difficulty walking, the use of walking aids, or pain while walking influenced how far they could walk. The individual’s motivation and energy levels also determined if and when the walk would take place. Strategies to address these challenges included adjusting the length of the walk, choosing the appropriate time of day, consulting their physiotherapist or the rehabilitation team about aids to facilitate walking, and tailoring the amount of conversation to each individual’s preferences during the walk.

*There is a nice gravel path where you can take those who have the best balance and like it. And those with more difficulties*,* then you take a slightly shorter walk and maybe sit somewhere. (I7)*

#### Motivation and guidance

The informants emphasized the importance of maintaining a positive attitude, not being afraid, and not giving up. They stressed the value of learning from both their own and their colleagues’ experiences of what worked and what did not. They recommended staying calm, happy, and patient, as stress could be contagious. Various methods to motivate residents to walk included enticing them with a short walk, nice weather, or the promise of coffee. It was noted that the approach to suggesting a walk was crucial; for some residents, it was more effective to avoid asking and simply say, ‘Come.’ For others, using gestures or showing outerwear signaled the plan. Importantly, no one was forced to go out—their wishes were respected. However, residents who were initially hesitant often changed their minds once they were outside. It was also important to be ready and dressed, as the resident’s desire to go out could change quickly.

*Yeah*,* I see a little glimpse or something. Which means that*,* yes*,* you can probably get them out*,* a while anyway. And then we go for a normal walk anyway*,* and they say: ‘Yes*,* it felt good’. (I3)*

Risks mentioned included residents becoming aggressive, wanting to go in a different direction, seeking to take a bus or tram, or refusing to return home. Staff managed these situations by diverting attention with the surroundings or discussing what was at home. Avoiding arguments and leveraging experience in dementia care were considered helpful in motivating residents and guiding them effectively during walks.

*One simply has to think a little*,* and not go too far*,* and maybe not go to a place where they have lived before. And stay calm. (I3)*

### Main category 3: Emotional, physical and relational responses to outdoor walks

Several benefits of walks for the residents were observed, including improvements in physical, psychological, cognitive, social, and existential well-being. However, in some cases walks were followed by increased anxiety. For staff, the walks offered advantages such as getting fresh air, physical activity during work hours, and providing meaningful activities. Yet, these positives were sometimes accompanied by feelings of stress and inadequacy. Relatives of the residents expressed appreciation for the opportunity their family members had to spend time outdoors. This recognition was described as rewarding for staff and contributed to a sense of purpose and value in their work.

#### Benefits and emotional shifts in residents

Informants observed emotional, physical and cognitive responses before, during and after the walks, such as better appetite, better sleep and improved walking ability. These changes were noted by all informants, regardless of the length of their work experience. Two informants with over 20 years of experience specifically reported having witnessed the positive effects of walks repeatedly over the years.

*With walking*,* like I already said*,* you don’t need to medicate anyone. They eat better. They can even sleep better too. (I5)*

*One resident*,* he hurt his hip a while ago and couldn’t walk […] it became a fun activity for him to get out. He kind of got his physical [ability] back in the leg. Today he can walk. It’s almost like me running after him. (I3)*

The social significance of the walks was described as fostering relationships with the residents, building trust, and providing a private space for sharing thoughts without the risk of being overheard. It was perceived that residents spoke more openly and clearly during walks, and that the walks often triggered memories, which could be seen as a form of cognitive stimulation. In some cases, residents were able to learn their way around and recognize staff with whom they frequently walked.

*For example*,* we have a lady who has very severe aphasia*,* and I have noticed that when you go for a walk she talks*,* it becomes easier to talk. She expresses herself in a way that you can understand what she means. (I1)*

All informants reported several positive effects on residents’ well-being. During and after the walks, residents appeared calmer and expressed emotions such as joy, gratitude, anticipation, and enjoyment. After the walk, staff observed reduced anxiety, aggression, and motor restlessness. In some dementia homes, the introduction of walking routines was associated with a measurable decrease in BPSD.

*She was anxious and then we went out. She was so happy. She forgot all this anxiety and worry that she had. (I5)*

*We do an evaluation [of BPSD scores]. We follow them over time*,* and we’ve seen a decrease*,* like*,* the graphs have gone down for them. So that’s basically what we use to measure it*,* so to speak. (I3)*

Residents also expressed a sense of freedom and found meaning in their day through walking, which was sometimes used as an alternative to sedative medications.

*It reduces anxiety. Reduces the need for as-needed medication. And maybe helps get a better daily rhythm. (I7)*

*Walks are really important. It can really be an alternative to sedatives. (I1)*

Few negative effects were reported. Only one informant noted that some residents might experience anxiety following the walks.

*And then there are those who also get very worried after a walk. You think it’s been really nice when you’ve been out and then when you get back home they go: ‘Oh my god*,* where have I been today?’ and ‘I was on my way somewhere’… And all of a sudden a thousand thoughts are set in motion and they can’t put it together. (I4)*

#### Ambivalent experiences among staff

The informants described an ambivalent experience, balancing the rewarding aspects of outdoor walks − for both residents and themselves − with feelings of stress. They valued the opportunity to get fresh air, be physically active, and spend time outdoors during their workday, often feeling less stressed and more energized afterward. The walks were also described as enjoyable and fulfilling, offering a meaningful sense of accomplishment.

*I mean*,* one of the upsides is that*,* as staff*,* you actually get to go outside during work hours*,* and I think that’s a great thing. (I6)*

*…and from relatives too: ‘Thank you so much! I heard that my mom got to come out with you*,* had a coffee and felt so good. She told me about it like a thousand times!’ So that kind of appreciation really means a lot too. (I2)*

It was suggested that beyond the immediate benefits, such as reduced anxiety among residents, regular walks could also reduce staff workload over time by helping residents remain physically mobile. However, the walks could also cause stress and feelings of inadequacy, particularly when leaving colleagues alone in their departments. Informants noted that some colleagues felt anxious or unsure about walking with residents with dementia, fearing they might stray or not knowing how to guide them back.

*But preferably a little after lunch. So that there are two staff on site. Because sometimes it can feel a bit rude if you go out with one person and then you leave your colleague with the rest. (I4)*

#### Emotional relief for family members

Family members’ responses were also highlighted. It was noted that residents’ family members sometimes experienced feelings of guilt about having their loved ones in a nursing home and therefore felt happiness and relief when their family members had the opportunity to go outdoors for walks.

*We have a bulletin board for the activities and outings we have. Sometimes I take pictures and put them up*,* so relatives can see […] Then they are very happy. ‘Oh*,* mom has the opportunity to go outside’. (I2)*

## Discussion

To our knowledge, this is the first study to explore how outdoor walks can be integrated into routine dementia care from the perspective of nursing staff, outside the context of structured interventions or research projects. The findings show that such walks are consistently perceived as meaningful and beneficial for residents’ well-being, while also revealing practical challenges and facilitators related to their implementation in everyday care. The perceived benefits, described by all informants, may be attributed to various factors, including physical activity, time spent outdoors in nature, engagement in meaningful activities, social interaction with staff, and change of environment. A person-centered approach was described as essential for achieving beneficial responses and facilitating implementation.

All informants noted that walks had a calming effect and could reduce anxiety and motor restlessness. Similar findings have been shown in intervention studies on the effects of physical activity on BPSD, although the literature remains inconclusive. One meta-analysis found that physical exercise reduced depression and motor restlessness but had a limited impact on apathy and agitation [34]. Another review noted that physical activity, including walking, positively influenced mood, depression, and agitation [35]. Unexpectedly, our study found that the walks sometimes could increase anxiety immediately afterward, even when the person seemed to enjoy the walk. One informant suggested introducing routines to offer walks before anticipated anxiety, particularly in the afternoon when sundown syndrome is common. Exposure to daylight and regular walks, especially in the morning or afternoon, have shown beneficial effects on sundown syndrome [28].

Informants also described how walking improved appetite and sleep. Although exercise is known to affect appetite regulation [36], it is less studied in dementia. Previous exercise interventions have not shown any significant effects on nutritional status or appetite [34, 37]. However, walking can improve bowel function and mood, indirectly stimulating appetite. Daily or frequent physical activity has also been shown to enhance sleep quality and quantity in people with dementia, with walking specifically associated with improved sleep in some studies [27, 28, 38].

Another reported response was that individuals with speaking difficulties were able to speak more clearly during, and sometimes after, the walk, suggesting cognitive effects. Direct effects on cognition have been seen after a single brief session of physical activity in healthy older adults [39], and exercise has shown improved cognition in people with dementia [11, 12]. Informants observed more conversations during the walks, possibly because social interaction may be less demanding outdoors, and natural environments stimulate memory by providing a link to the past and memories of childhood, facilitating engagement [40]. Improved physical function was also mentioned, consistent with the well-known benefits of physical activity or exercise in general [8], as well as findings from earlier studies involving people with dementia [11].

Challenges to integrating walks into daily routines, included weather and residents’ varying individual conditions, such as pain, fall risk, fatigue, and reduced motivation. Adaptive strategies, such as using blankets, bringing drinks, avoiding slippery paths, selecting quiet routes, or opting for indoor walks helped address these challenges. Walks were tailored to individual needs by adjusting timing and distance, or using a wheelchair, highlighting the need for dementia-friendly environments to support walking, in line with Barrett et al. [41], who identified weather and lack of outdoor amenities as limiting access to nature for residents with dementia.

National dementia care guidelines emphasize person-centered care that supports the whole person, helping each individual maximize their abilities and remain socially engaged [7]. Though informants did not use the term “person-centered” explicitly, they described tailoring walks to individual needs, such as timing, preferred destinations, communication methods, and known triggers to avoid conflict. They adjusted walk duration and conversation levels as needed. Walks provided unique opportunities for social connection through focused, private conversations, reflecting core person-centered principles of individualized attention and relational closeness. While person-centered physical activity in dementia care is underexplored, similar benefits have been reported when walking activities are tailored to individual preferences and informed by background information from relatives, emphasizing meaningful one-on-one engagement [24, 42]. The observed outcomes, improvements in physical function, behavior, communication and mood, align well with those described by informants in our study.

Walking with people living with dementia differs from somatic care due to reduced initiative and apathy, which often make residents reliant on staff to initiate physical activity [17]. Several informants noted that residents initially reluctant to go out often enjoyed the walk once outside, though they emphasized never forcing anyone. Various strategies were used to motivate and guide residents during walks, such as maintaining a calm, positive tone, highlighting pleasant weather or fresh air, and offering small incentives like a cup of coffee, and reassuring that the walk would be short. If residents resisted following the intended route, staff diverted their attention by discussing the surroundings or simply changing the subject. Avoiding arguments was key to preventing conflict.

Nature exposure positively affects health and well-being [19], and people with Alzheimer’s appreciate outdoor activities [21]. However, most dementia research has focused on gardening [20, 43] and walking studies in nursing homes have often focused on indoor activity [44]. This study adds new insights into the benefits of outdoor walking for this population.

Informants also noted that walking had positive effects on their own well-being, consistent with Evans et al. [45], who suggested that outdoor activities with dementia patients increased staff job satisfaction and reduced sickness absence. Positive feedback from the residents’ relatives was also mentioned as rewarding. They valued the opportunity for their family members to spend time outdoors and go for walks. It is known that having a family member living in a nursing home can affect relatives in various ways, evoking feelings of powerlessness and guilt [46].

However, they also expressed frustration at not being able to offer outdoor walks as often as desired, despite recognizing their clear benefits, due to time constraints, staff shortages, and lack of routines. Since outdoor walks often require one-to-one staff support, planning for sufficient staffing is essential, not only to enable walks but also to ensure that other residents’ needs are met. A person-centered approach demands flexibility. Previous research has similarly emphasized the need for one-on-one accompaniment and identified limited staff availability as a key barrier [42, 43]. Informants also raised concerns about the absence of legal recognition of outdoor access rights for people with dementia, reflecting a broader human rights perspectives. A 2023 survey by the Swedish National Board of Health and Welfare reported that 26% of care home residents rated their opportunities to go outdoors as bad or very bad [47].

Varying attitudes among colleagues regarding the importance of outdoor walks were described. Some staff who prioritized walks were described as “enthusiasts,” while others viewed them as neglecting other duties like cleaning. Informants reflected that colleagues who are less interested in being outdoors themselves may also be less inclined to take residents out for walks. This aligns previous findings identifying staff knowledge, attitudes, and a task-oriented, non-person-centered care culture as barriers to outdoor activity [41]. MacAndrew et al. [27] described initial staff hesitation, which shifted as residents expressed enjoyment and staff–resident relationships improved, facilitating daily care. To implement walking into routine dementia care, staff need enhanced knowledge of its benefits, awareness of potential risks (e.g., falls, confusion, anxiety) and strategies to mitigate them, as well as insight into how dementia impacts initiative and contributes to apathy. In addition, support and guidance from management are essential.

### Strengths and limitations

Despite some limitations, this study is unique in exploring outdoor walks as part of routine dementia care from nursing staff’s viewpoint. Strengths include the informants’ extensive dementia care knowledge and experience (3–22 years), diverse settings (urban and rural), variation in age, education, and ethnicity, offering rich perspectives and valuable insights. However, recruitment challenges limited the sample size and its diversity, as all were women with positive attitudes toward outdoor walks, which may limit transferability. Some informants also described themselves as individuals who enjoyed nature and being outside. This personal interest, along with their strong commitment to the residents and to creating meaningful walking experiences, may have influenced their perception of the walks’ positive effects. It is possible that the findings would have differed if the staff had demonstrated a lower level of interest and engagement. Despite the small and relatively homogenous sample, it can be argued that sufficient information power was achieved, given the study’s focused aim, the specificity and experience of the participants, and the richness of the data collected [48].

The interviewer (first author) had specific experience working in dementia care homes, which provided an understanding of the informants’ experiences and context, thereby facilitating the interview process. The co-authors’ expertise in qualitative methods, research on health-enhancing physical activity in older adults, and dementia prevention strengthened the analysis, as researchers’ experiences, qualifications, and training significantly influence data interpretation [48]. Nevertheless, prior understanding could also overshadow new insights and limit the recognition of all relevant data.

While acknowledging the importance of including people with dementia in research, this study did not include the residents’ own perceptions of outdoor walks as part of their routine care, which may be seen as a limitation. Existing research on experiences with outdoor activities and physical activity has largely focused on individuals in the early stages of dementia [21, 22]. Future studies should explore the subjective experiences of those with moderate to severe impairment.

## Conclusions

Despite the health benefits of daily physical activity and the belief that outdoor access is a human right, there is no law guaranteeing daily outdoor time for individuals with dementia in Sweden. This study found that outdoor walks were valuable for residents in dementia care homes, staff, and relatives from multiple perspectives. Outdoor walks were perceived to enhance well-being, provide a sense of meaning, foster social interaction, and connect residents with nature and the community. Informants regarded walks as important and described beneficial effects on both physical and mental health, including BPSD, suggesting that walks could potentially reduce the need for medication.

Implementing walks in dementia care homes involves challenges related to legislation, organization, environment, weather, and individual factors. Knowledge and experience in dementia care helped in managing these challenges. Informants described a person-centered approach to planning and conducting walks, tailored to each resident’s physical and cognitive abilities, mood, interests, and communication style. Despite limitations such as lack of time, staffing, and routines, and the inherent difficulties of dementia, informants valued walks highly and found various ways to integrate them into daily care.

## Supplementary Information

Below is the link to the electronic supplementary material.

Supplementary Material 1

## Data Availability

Raw de-identified data may be made available upon reasonable request from the corresponding author.

## References

[1] Nichols E, Steinmetz JD, Vollset SE, Fukutaki K, Chalek J, Abd-Allah F, et al. Estimation of the global prevalence of dementia in 2019 and forecasted prevalence in 2050: an analysis for the global burden of disease study 2019. Lancet Public Health. 2022;7(2):e105–25. 34998485 10.1016/S2468-2667(21)00249-8 PMC8810394

[2] Frisell O, Jönsson L, Wimo A. Demenssjukdomarnas Samhällskostnader i Sverige 2019. Karolinska Institutet; 2019.

[3] Budson A. Kowall, N. The handbook of alzheimer´s disease and other dementias. Wiley Blackwell; 2014.

[4] Sverdrup K, Selbæk G, Bergh S, Strand BH, Thingstad P, Skjellegrind HK, et al. Physical performance across the cognitive spectrum and between dementia subtypes in a population-based sample of older adults: the HUNT study. Arch Gerontol Geriatr. 2021;95:104400. 33798998 10.1016/j.archger.2021.104400

[5] Van Doorn C, Gruber-Baldini AL, Zimmerman S, Richard Hebel J, Port CL, Baumgarten M, et al. Dementia as a risk factor for falls and fall injuries among nursing home residents. J Am Geriatr Soc. 2003;51(9):1213–8. 12919232 10.1046/j.1532-5415.2003.51404.x

[6] Kales HC, Gitlin LN, Lyketsos CG. Assessment and management of behavioral and psychological symptoms of dementia. BMJ. 2015;350:h369. 25731881 10.1136/bmj.h369 PMC4707529

[7] Socialstyrelsen. Nationella Riktlinjer för vård och Omsorg vid demenssjukdom. Åtta.45 tryckeri AB. The National Board of Health and Welfare. 2017.

[8] World Health Organisation. Dementia 2023. Available from: https://www.who.int/news-room/fact-sheets/detail/dementia

[9] Blankevoort CG, van Heuvelen MJ, Boersma F, Luning H, de Jong J, Scherder EJ. Review of effects of physical activity on strength, balance, mobility and ADL performance in elderly subjects with dementia. Dement Geriatr Cogn Disord. 2010;30(5):392–402. 20980758 10.1159/000321357

[10] Zhou S, Chen S, Liu X, Zhang Y, Zhao M, Li W. Physical activity improves cognition and activities of daily living in adults with alzheimer’s disease: a systematic review and meta-analysis of randomized controlled trials. Int J Environ Res Public Health. 2022;19(3):1216. 35162238 10.3390/ijerph19031216 PMC8834999

[11] Demurtas J, Schoene D, Torbahn G, Marengoni A, Grande G, Zou L et al. Physical activity and exercise in mild cognitive impairment and dementia: an umbrella review of intervention and observational studies. J Am Med Dir Assoc. 2020;21(10):1415-22.e6. 10.1016/j.jamda.2020.08.031 32981668

[12] Venegas-Sanabria LC, Martínez-Vizcaino V, Cavero-Redondo I, Chavarro-Carvajal DA, Cano-Gutierrez CA, Álvarez-Bueno C. Effect of physical activity on cognitive domains in dementia and mild cognitive impairment: overview of systematic reviews and meta-analyses. Aging Ment Health. 2021;25(11):1977–85. 33143444 10.1080/13607863.2020.1839862

[13] Zeng Y, Wang J, Cai X, Zhang X, Zhang J, Peng M, et al. Effects of physical activity interventions on executive function in older adults with dementia: A meta-analysis of randomized controlled trials. Geriatr Nurs. 2023;51:369–77. 37127013 10.1016/j.gerinurse.2023.04.012

[14] Winchester J, Dick MB, Gillen D, Reed B, Miller B, Tinklenberg J, et al. Walking stabilizes cognitive functioning in alzheimer’s disease (AD) across one year. Arch Gerontol Geriatr. 2013;56(1):96–103. 22959822 10.1016/j.archger.2012.06.016 PMC3766353

[15] Hanson S, Jones A. Is there evidence that walking groups have health benefits? A systematic review and meta-analysis. Br J Sports Med. 2015;49(11):710–5. 25601182 10.1136/bjsports-2014-094157 PMC4453623

[16] Hartman YAW, Karssemeijer EGA, van Diepen LAM, Olde Rikkert MGM, Thijssen DHJ. Dementia patients are more sedentary and less physically active than age- and sex-matched cognitively healthy older adults. Dement Geriatr Cogn Disord. 2018;46(1–2):81–9. 30145584 10.1159/000491995 PMC6187840

[17] van Alphen HJ, Volkers KM, Blankevoort CG, Scherder EJ, Hortobágyi T, van Heuvelen MJ. Older adults with dementia are sedentary for most of the day. PLoS ONE. 2016;11(3):e0152457. 27031509 10.1371/journal.pone.0152457 PMC4816298

[18] Gibson G, Chalfont GE, Clarke PD, Torrington JM, Sixsmith AJ. Housing and connection to nature for people with dementia. J Hous Elder. 2007;21(1–2):55–72.

[19] Gagliardi C, Piccinini F. The use of nature - based activities for the well-being of older people: an integrative literature review. Arch Gerontol Geriatr. 2019;83:315–27. 31128876 10.1016/j.archger.2019.05.012

[20] van der Velde-van Buuringen M, Hendriks-van der Sar R, Verbeek H, Achterberg WP, Caljouw MAA. The effect of garden use on quality of life and behavioral and psychological symptoms of dementia in people living with dementia in nursing homes: a systematic review. Front Psychiatry. 2023;14:1044271. 37124273 10.3389/fpsyt.2023.1044271 PMC10130442

[21] Cedervall Y, Torres S, Åberg AC. Maintaining well-being and selfhood through physical activity: experiences of people with mild alzheimer’s disease. Aging Ment Health. 2015;19(8):679–88. 25265932 10.1080/13607863.2014.962004

[22] McDuff J, Phinney A. Walking with meaning:subjective experiences of physical activity in dementia. Glob Qual Nurs Res. 2015;2:2333393615605116. 28462316 10.1177/2333393615605116 PMC5342298

[23] Duggan S, Blackman T, Martyr A, Van Schaik P. The impact of early dementia on outdoor life: A `shrinking world’? Dementia. 2008;7(2):191–204.

[24] Chu CH, Puts M, Brooks D, Parry M, McGilton KS. A feasibility study of a multifaceted walking intervention to maintain the functional mobility, activities of daily living, and quality of life of nursing home residents with dementia. Rehabil Nurs. 2020;45(4):204–17. 30325875 10.1097/rnj.0000000000000186

[25] Littbrand H, Stenvall M, Rosendahl E. Applicability and effects of physical exercise on physical and cognitive functions and activities of daily living among people with dementia: a systematic review. Am J Phys Med Rehabil. 2011;90(6):495–518. 21430516 10.1097/PHM.0b013e318214de26

[26] Venturelli M, Scarsini R, Schena F. Six-month walking program changes cognitive and ADL performance in patients with alzheimer. Am J Alzheimers Dis Other Demen. 2011;26(5):381–8. 21852281 10.1177/1533317511418956 PMC10845333

[27] MacAndrew M, Kolanowski A, Fielding E, Kerr G, McMaster M, Wyles K, et al. ″Would you like to join me for a walk?″ The feasibility of a supervised walking programme for people with dementia who wander. Int J Older People Nurs. 2019;14(3):e12244. 31125189 10.1111/opn.12244

[28] Shih Y-H, Pai M-C, Lin H-S, Sung P-S, Wang J-J. Effects of walking on sundown syndrome in community-dwelling people with alzheimer’s disease. Int J Older People Nurs. 2020;15(2):e12292. 31814316 10.1111/opn.12292

[29] Patton MQ. Qualitative Research & Evaluation Methods 3rd edition. Thousand Oaks, CA: SAGE publication Inc; 2002.

[30] Elo S, Kyngas H. The qualitative content analysis process. J Adv Nurs. 2008;62(1):107–15. 18352969 10.1111/j.1365-2648.2007.04569.x

[31] Lindgren BM, Lundman B, Graneheim UH. Abstraction and interpretation during the qualitative content analysis process. Int J Nurs Stud. 2020;108:103632. 32505813 10.1016/j.ijnurstu.2020.103632

[32] Elo S, Kääriäinen M, Kanste O, Pölkki T, Utriainen K, Kyngäs K. H. Qualitative content analysis: a focus on trustworthiness. SAGE Open. 2014;January–March:1–10.

[33] Tong A, Sainsbury P, Craig J. Consolidated criteria for reporting qualitative research (COREQ): a 32-item checklist for interviews and focus groups. Int J Qual Health Care. 2007;19(6):349–57. 17872937 10.1093/intqhc/mzm042

[34] de Souto Barreto P, Demougeot L, Pillard F, Lapeyre-Mestre M, Rolland Y. Exercise training for managing behavioral and psychological symptoms in people with dementia: A systematic review and meta-analysis. Ageing Res Rev. 2015;24(Pt B):274–85. 26369357 10.1016/j.arr.2015.09.001

[35] Brett L, Traynor V, Stapley P. Effects of physical exercise on health and well-being of individuals living with a dementia in nursing homes: a systematic review. J Am Med Dir Assoc. 2016;17(2):104–16. 26432622 10.1016/j.jamda.2015.08.016

[36] Dorling J, Broom DR, Burns SF, Clayton DJ, Deighton K, James LJ et al. Acute and chronic effects of exercise on appetite, energy intake, and appetite-related hormones: the modulating effect of adiposity, sex, and habitual physical activity. Nutrients. 2018;10(9). 10.3390/nu10091140 PMC6164815 30131457

[37] Sampaio A, Marques-Aleixo I, Seabra A, Mota J, Carvalho J. Physical exercise for individuals with dementia: potential benefits perceived by formal caregivers. BMC Geriatr. 2021;21(1):6. 33407194 10.1186/s12877-020-01938-5 PMC7789403

[38] Richards KC, Lambert C, Beck CK, Bliwise DL, Evans WJ, Kalra GK, et al. Strength training, walking, and social activity improve sleep in nursing home and assisted living residents: randomized controlled trial. J Am Geriatr Soc. 2011;59(2):214–23. 21314643 10.1111/j.1532-5415.2010.03246.x PMC3124380

[39] Erickson KI, Hillman C, Stillman CM, Ballard RM, Bloodgood B, Conroy DE, et al. Physical activity, cognition, and brain outcomes: a review of the 2018 physical activity guidelines. Med Sci Sports Exerc. 2019;51(6):1242–51. 31095081 10.1249/MSS.0000000000001936 PMC6527141

[40] Bennett J, Wolverson E, Price E. Me, myself, and nature: living with dementia and connecting with the natural world - more than a breath of fresh air? A literature review. Dementia (London). 2022;21(7):2351–76. 10.1177/14713012221117896 35939420

[41] Barrett J, Evans S, Mapes N. Green dementia care in accommodation and care settings: a literature review. Hous Care Support. 2019;22(4):193–206.

[42] Chu CH, Quan AML, Gandhi F, McGilton KS. Perspectives of substitute decision-makers and staff about person‐centred physical activity in long‐term care. Health Expect. 2022;25(5):2155–65. 34748256 10.1111/hex.13381 PMC9615080

[43] Whear R, Coon JT, Bethel A, Abbott R, Stein K, Garside R. What is the impact of using outdoor spaces such as gardens on the physical and mental well-being of those with dementia? A systematic review of quantitative and qualitative evidence. J Am Med Dir Assoc. 2014;15(10):697–705. 25037168 10.1016/j.jamda.2014.05.013

[44] Barrett E, Casey B, Dollard M, McCarthy B, Casey D. Effectiveness of functionally based physical activity programs on physical, psychological, cognitive, and adverse outcomes in older adults living in nursing homes: systematic review. Act Adapt Aging. 2021;45(4):306–47.

[45] Evans SC, Barrett J, Mapes N, Hennell J, Atkinson T, Bray J, et al. Connections with nature for people living with dementia. Work Older People. 2019;23(3):142–51.

[46] Dorell Å, Sundin K. Expressed emotions and experiences from relatives regarding having a family member living in a nursing home for older people. SAGE Open Med. 2019;7:2050312118823414. 30671245 10.1177/2050312118823414 PMC6329026

[47] Socialstyrelsen. Vad tycker de äldre om äldreomsorgen? The National Board of Health and Welfare. 2023;2023-10-8759.

[48] Malterud K. Qualitative research: standards, challenges, and guidelines. Lancet. 2001;358(9280):483–8. 11513933 10.1016/S0140-6736(01)05627-6

