# Supplementary material for: Finding the way - nursing staff’s perceptions and experiences of outdoor walks with residents in dementia care homes: a qualitative study
Source: BMC Nurs. 2025 Sep 1;24:1141. doi: 10.1186/s12912-025-03771-w (PMC12403861; doi:10.1186/s12912-025-03771-w)
Supplement: Supplementary file 1 — Supplementary Material 1 [file 12912_2025_3771_MOESM1_ESM.docx]

**Appendix – Interview guide***

Thank you for taking the time to participate in this interview study.

The purpose of this project is to describe the experiences and perceptions of nursing staff regarding outdoor walks with residents in dementia care facilities. I would therefore like to learn from your experiences of using outdoor walks for people with dementia. By "walks," I mean when individuals go outside, either with or without assistive devices.

All your experiences, opinions, and thoughts are valuable, and there are no right or wrong answers.

I will record the interview, and the content may be presented in research reports, but it will not be possible to identify who you are or who said what. Once I have completed the transcription and analysis of the interviews, the recordings will be deleted. Please try to minimize background noise, such as tapping on the table.

Of course, you only answer and discuss the questions you feel comfortable with, and you can stop the interview at any time.
Do you have any questions before we begin?

**INTERVIEW QUESTIONS**

**Can you tell me about your experience of taking outdoor walks with people with dementia living in the care facility where you work?**

**How do you incorporate walks into your work at the facility?**

- How do you think it works?

**What would you say is the purpose of the walks?**

- Are there multiple purposes?
- Do your colleagues share the same view?

**How do you plan outdoor walks? (**for yourself, for the resident, considering the environment)

**What difficulties exist in carrying out walks?** (yourself, the resident, the environment)

- How do you manage these difficulties?

**Are there any risks, and if so, what are they?**

- How do you handle potential risks?

**What facilitates the implementation of walks?** (yourself, the resident, the environment)

**What benefits or effects do you see from the walks? (**during and after the walks)

- How do you experience the walks?
- How do the residents experience the walks?

**Do you have any advice or tips for others who want to implement outdoor walks for people with dementia?**

- Something you have learned from your experiences?

**Is there anything we haven’t discussed regarding walks that you would like to bring up?**

- Anything else you would like to add?

**Thank you for sharing your experiences!**

**Follow-up prompts:**

Can you tell me more about that? Can you elaborate?

Have I understood you correctly…? Do you mean…?

How do you think about that? Can you give an example?

*Chat GPT-4 was first used for translation of the guide (2025-03-18) and then revised and checked by authors IMD and PG.
